# Deciphering the distance to antibiotic resistance for the pneumococcus using genome sequencing data

**DOI:** 10.1038/srep42808

**Published:** 2017-02-16

**Authors:** Fredrick M. Mobegi, Amelieke J. H. Cremers, Marien I. de Jonge, Stephen D. Bentley, Sacha A. F. T. van Hijum, Aldert Zomer

**Affiliations:** 1 Laboratory of Pediatric Infectious Diseases, Radboud Institute for Molecular Life Sciences, Radboud University Medical Centre, Nijmegen 6525 GA, The Netherlands; 2 Center for Molecular and Biomolecular Informatics, Radboud Institute for Molecular Life Sciences, Radboud University Medical Centre, Nijmegen 6525 GA, The Netherlands; 3 Division of Molecular Carcinogenesis, The Netherlands Cancer Institute, Amsterdam 1066 CX, The Netherlands; 4 The Wellcome Trust Sanger Institute, Wellcome Trust Genome Campus, Hinxton, Cambridge CB10 15A, UK; 5 Faculty of Veterinary Medicine, Department of Infectious Diseases and Immunology, Utrecht University, Utrecht 3508 TD, The Netherlands

## Abstract

Advances in genome sequencing technologies and genome-wide association studies (GWAS) have provided unprecedented insights into the molecular basis of microbial phenotypes and enabled the identification of the underlying genetic variants in real populations. However, utilization of genome sequencing in clinical phenotyping of bacteria is challenging due to the lack of reliable and accurate approaches. Here, we report a method for predicting microbial resistance patterns using genome sequencing data. We analyzed whole genome sequences of 1,680 *Streptococcus pneumoniae* isolates from four independent populations using GWAS and identified probable hotspots of genetic variation which correlate with phenotypes of resistance to essential classes of antibiotics. With the premise that accumulation of putative resistance-conferring SNPs, potentially in combination with specific resistance genes, precedes full resistance, we retrogressively surveyed the hotspot loci and quantified the number of SNPs and/or genes, which if accumulated would confer full resistance to an otherwise susceptible strain. We name this approach the ‘distance to resistance’. It can be used to identify the creep towards complete antibiotics resistance in bacteria using genome sequencing. This approach serves as a basis for the development of future sequencing-based methods for predicting resistance profiles of bacterial strains in hospital microbiology and public health settings.

*Streptococcus pneumoniae*, or the pneumococcus, is part of the normal bacterial flora of the human nasopharynx, but can occasionally infiltrate sterile sites of the body progressing to disease. An estimated 1.6 million deaths associated with *S. pneumoniae* are reported every year worldwide, mostly affecting children under five years. Despite the immunization efforts reducing pneumococcal disease, there is merely a marginal prospect of eliminating pneumococcal disease because the available pneumococcal conjugate vaccines (PCV) only protect against 13 of the over 97 circulating serotypes. Removal of vaccine-types also leads to rapid serotype replacement that consequently increases carriage prevalence and disease by non-vaccine serotypes with occasional increase in antibiotic resistance^1^.

Since the first case of penicillin-resistant pneumococcus was reported^2^, followed by outbreaks of disease caused by multidrug-resistant pneumococci^3^, the antibiotic resistance patterns of *S. pneumoniae* have drastically evolved and escalated worldwide. The pneumococcus is known to be highly recombinogenic allowing sequences that confer antimicrobial non-susceptibility to be readily introduced into the genome. Discovery of the genetic determinants underlying microbial phenotypes such as antimicrobial resistance and virulence is an important question in microbiology. Traditionally, changes in DNA which are associated with antibiotic resistance in *S. pneumoniae* were identified using sequence comparison, laboratory mutagenesis^4^, and identification of horizontally transferred sequences^5^. These techniques are limited in specificity and sensitivity necessary for applications in clinical laboratories. They only reveal common genomic regions where change has occurred in the so-called ‘mosaic’ genes, are narrow in their application to study actual populations, and may miss out on situations where multiple mutations occurring in different genomic loci are required for full antibiotic resistance^5^,^6^. Significant advances in high-throughput genome sequencing technologies and bacterial genome-wide association studies (GWAS) now allow identification of statistical association between plausible causal genetic variants and microbial phenotypes in real populations^7^. This approach was recently used to identify the single nucleotide polymorphisms (SNPs) in DNA that may confer beta-lactam resistance in *S. pneumoniae*^6^.

However, understanding how genetic variations contribute to antibiotics resistance remains underexplored. Here we report the use of genome sequencing and GWAS to investigate SNPs and genes associated with resistance to four essential classes of antibiotics; collectively referred to as antibiotic resistance hereafter. We name the cumulative effect of these resistance-conferring variants the “distance to resistance” for the pneumococcus. We analyzed 1,680 invasive and carriage pneumococcal isolates from Nijmegen, the Netherlands^8^, Massachusetts, USA^9^, Maela, Thailand^6^, and isolates from Sickle-cell anemic children (henceforth referred to as SCD; sickle cell disease) in the USA^10^. The genotypic and phenotypic diversity in these independent cohorts, whose draft genomes and phenotypes for antibiotic resistance are available, represent a unique dataset for identifying the contribution of each putative antibiotic resistance-conferring genetic variant to the pneumococcal resistance profile. With the premise that presence of particular genes or accumulation of specific SNPs precedes full drug resistance of a fit clone, whole genome sequencing and GWAS could be used to evaluate the rate of accumulation of candidate resistance-conferring variants and provide an early warning sign of increasing antibiotic resistance. We hypothesize that the SNPs and/or resistance associated genes separating the phenotypes of antibiotic resistance are the plausible maximum number of mutations, which if accumulated could render high antibiotic resistance to otherwise susceptible bacteria. As the clinics gradually embrace genome sequencing for microbiological analyses, the ability to use genomic sequencing data to predict relevant phenotypes such as antibiotic resistance will be essential and desirable. This study serves as a foundation for the development of future technologies that could utilize genomic sequencing to analyze the molecular epidemiological trends for bacterial strains reliably, and provide an early-warning measure for the edge towards antimicrobial resistance, crucially informing on clinical intervention strategies.

## Results

### Pneumococcal strains and phenotypes of antibiotic resistance

**Figure 1:**
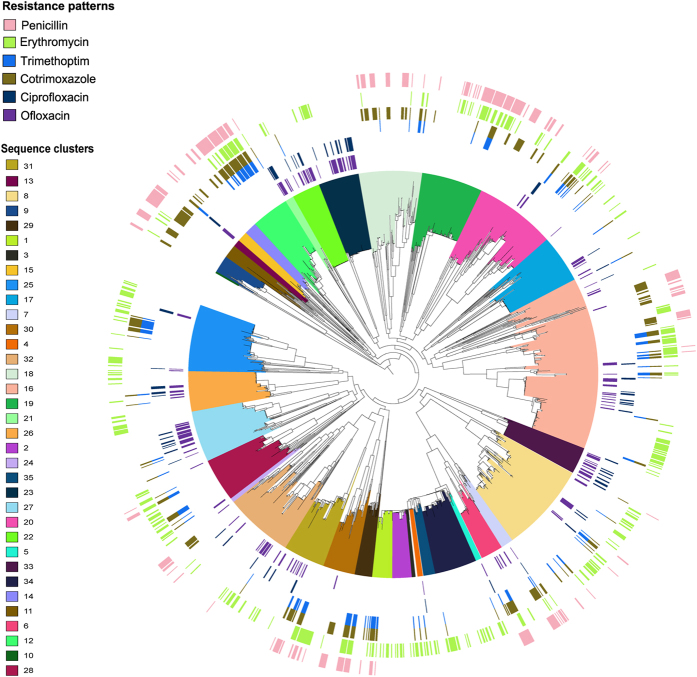
A maximum likelihood phylogeny of the concatenated variant regions from the core genome of 1682 pneumococcal isolates. The clades are colored according to the sequence clusters. The circular stripes represent the antibiotic resistance phenotypes starting from the outermost: pink; penicillin, green; erythromycin, blue; trimethoprim, gold; cotrimoxazole, navy-blue; ciprofloxacin, and purple; ofloxacin.

We analyzed 1,680 disease and carriage *S. pneumoniae* isolates systematically selected from diverse cohorts (see materials and methods). In carriage isolates from Maela and Massachusetts, 54 of the 1,012 were resistant to trimethoprim, penicillin, erythromycin and cotrimoxazole, representing about 0.054% resistance to four classes of essential antibiotics (multidrug resistance; MDR). Additionally, all 263 carriage isolates that showed full resistance to penicillin were also resistant to at least one other antibiotic of a different class (~26% resistance to penicillin and one other antibiotic). In contrast, only one isolate from Nijmegen (10208_2#41) showed resistance to all antibiotics tested. Three isolates from Nijmegen exhibited resistance to penicillin (Fig. 1; Supplementary Table S1).

Overall, the Nijmegen cohort had the lowest percentage proportion of isolates showing antibiotic resistance; penicillin 0.86% (3 isolates), trimethoprim 4.29% (15), erythromycin 2.28% (8), and cotrimoxazole 4.29% (15), as compared to the selection of Maela and Massachusetts isolates; penicillin 25.99% (263), trimethoprim 14.13% (143 isolates), erythromycin 35.57% (360), and cotrimoxazole 27.76% (281). However, the proportions of resistance to fluoroquinolones; ciprofloxacin 22.86% (80) and ofloxacin 50% (175) were remarkably high in the Nijmegen IPD isolates.

### Specific polymorphisms and genes showing significant association with antibiotic resistance

**Figure 2:**
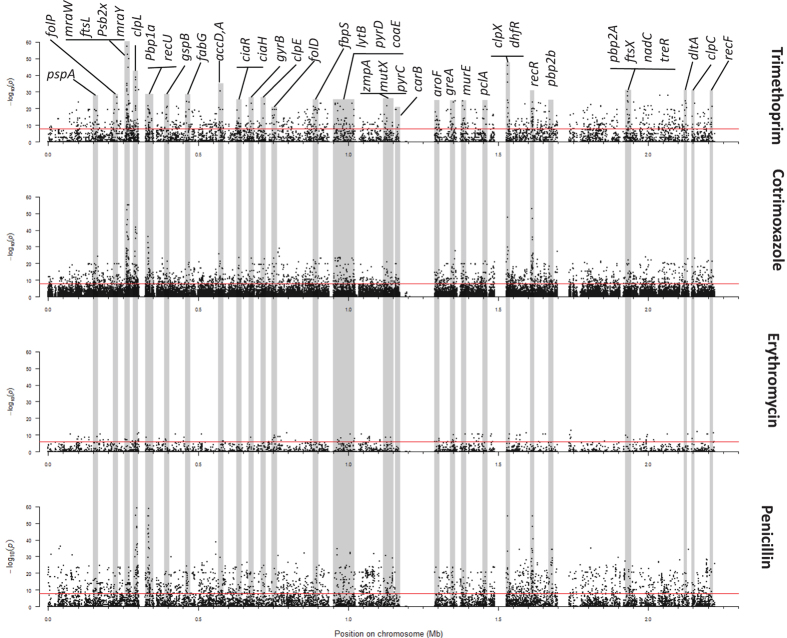
Manhattan plots summarizing the statistical significance of genome-wide associations between whole-genome SNPs and resistance to various antibiotics. Specific loci that are significantly associated with resistance to antibiotic are shown in the top panel.

Candidate putative causal variants were selected as SNPs showing statistically significant associations with the resistance phenotype (*p-values* < 0.01, stratified for population substructure and Bonferroni-adjusted for multiple testing). Separate associations were tested for resistance to penicillin, trimethoprim, cotrimoxazole, erythromycin, ciprofloxacin, and ofloxacin. We identified, 4,317 SNPs that confer resistance to penicillin, 3,589 in coding or intragenic sequences and 548 in non-coding or intergenic sequences (Fig. 2; Supplementary Table S2). A q-q plot of the penicillin GWAS *p-values* showed a sharp deviation above an expected *p*-value indicating the presence of unusually high linkage disequilibrium (LD) and strong association of the phenotype with SNPs in heavily genotyped loci. To further control for inflation and increase confidence in verity, a more stringent *p*-value threshold cut-off was applied at the point of deviation of the observed *p-values* from the expected *p*-values. This new cut-off (*p*-value < 1.5E-20) produced a smaller subset of 426 SNPs associating to the penicillin resistance phenotype. The SNPs are localized primarily in genes previously reported to be involved in development of penicillin resistance, including genes involved in the peptidoglycan biosynthesis pathway such as penicillin binding proteins; PBPs (*pbp1A, pbp1B, pbpX, pbp2A, penA*), peptidoglycan biogenesis transferases (*mraW, mraY*) and synthesis of peptidoglycan precursors (*murM, murN*), pneumococcal surface proteins (*pspA, pspC*), recombination pathway (*recU*), cell division pathway (*gpsB, ftsL*), multi-drug resistance (MDR) proteins and drug efflux pumps (*pmrA*), drug antiporters^6^, heat shock proteins/chaperones (ClpL)^11^, and genes implicated in resistance to other essential classes of antibiotics like dihydrofolate reductase (*dyr*); involved in trimethoprim resistance^12^,^13^.

We employed a machine learning method, Random Forest (RF), to investigate the combinatorial effect of certain SNPs and or/genes and prioritize causal variants. From the 426 SNPs, the RF model identified 34 unique SNPs that are predictive of penicillin resistance (Table 1). A separate GWAS on the presence or absence of individual genes in each isolate also revealed that presence of variants of the cell division initiation protein, *gpsB* (og_2891 and og_1645; *p-*values 4.73E-44 and 4.06E-43 respectively; Bonferroni-adjusted for multiple testing) significantly correlates with penicillin resistance. Combining both the SNPs and the genes (represented by orthologous groups; OGs) in a RF model further determined that the gene *gpsB* (og_2891 and og_1645) is the only gene among the top 20 features (SNPS and genes) that are predictive of penicillin resistance.

Penicillin-resistant pneumococcus exhibit varying patterns of resistance to other β-lactams and are generally resistant to other classes of antibiotics that are usually active against pneumococci^14^. We evaluated resistance to trimethoprim and cotrimoxazole, erythromycin, as well as ofloxacin and ciprofloxacin. We identified SNPs associated with resistance to trimethoprim and cotrimoxazole in various genes encoding enzymes involved in folate metabolism, including *dyr, folE*, and *folP* (Fig. 2; Supplementary Tables S3–S4), and SNPs in genes implicated in resistance to other essential antibiotics like penicillin. RF analysis revealed that only mutations in genes involved in folate metabolism (*dyr, folC, folE, folP*, and 2-amino-4-hydroxy-6-hydroxymethyldihydropteridine diphosphokinase; HPPK (EC 2.7.6.3) encoded by *folK or sulD*), and chaperones/ATP-dependent proteases (*clpL, clpX*)^15^, as well as linked mutations in PBPs (*pbp1A, pbpX, penA*), recombination proteins (*recR, recU*), and peptidoglycan biogenesis transferases (*mraW, mraY*), were predictive of cotrimoxazole and trimethoprim resistance (Tables 2 and 3).

Target site modification, characterized by presence of the *ermB* gene, and efflux from bacteria, mediated by the product of the *mefA* gene, are the most common mechanisms of bacterial resistance to macrolides. Before correcting for multiple testing, we observed a statistically significant association between the presence of *ermB* (og_1123) and resistance to erythromycin (*p*-value 3.137E-05). The OG comprises of two variants of the 23S rRNA (adenine [2058]-N6-)-methyltransferase (ermB): one variant is of the *Staphylococcus aureus* origin (ungapped protein blast - blastp - alignment with 100% sequence identity over 100% sequence coverage and an e-value of 3e-176). Surprisingly, this association significance diminishes after correcting for multiple testing (*p*-value 0.09; Bonferroni-corrected for multiple testing). Even so, we identified SNPs in the 16S rRNA; *rsmE*, 50S rRNA; *rplM*/*S*/*B*/*T, rpmA*/*E2*/*F*/*H, rplE*/L/I, and 30S rRNA; *rpsA*/*M*/*N*/*P*/*D*/*H* molecules that significantly associated with erythromycin resistance (Fig. 2; Supplementary Table S5). In the RF analysis, the presence of various genes was associated with resistance to erythromycin (Table 4). They include the macrolide efflux pump *mefA* (og_1312); ImpB/MucB/SamB family protein (og_1652): a family of error-prone DNA polymerases involved in DNA repair; the YolD-like protein (og_1379), a group of functionally uncharacterized proteins predicted to be functionally alike to the UmuD subunit of polymerase V from Gram-negative bacteria, and the ribose import ATP-binding protein (og_791; SP_1114).

Resistance in *S. pneumoniae* to fluoroquinolone is caused predominantly by mutations in DNA gyrase (*gyrA*) or DNA topoisomerase (*parC* and occasionally *parE*), which reduces binding of the drug to the site of activity. We did not observe any associations between SNPs in fluoroquinolone target proteins (DNA topoisomerase and DNA gyrase) and fluoroquinolone resistance (Supplementary Table S5). Nonetheless, we observed a statistically significant association between mutations in a multi-drug resistance efflux pump (*pmrA*) and resistance to ofloxacin (*p*-value 5.06E-05). We also observed that mutations in the heme exporter protein A (*ccmA*) and the recombination factor protein (*rarA*) associated with resistance to the fluoroquinolone ciprofloxacin.

### Predicting antibiotic resistance profiles and the prospective value of the ‘distance to resistance’

**Figure 3:**
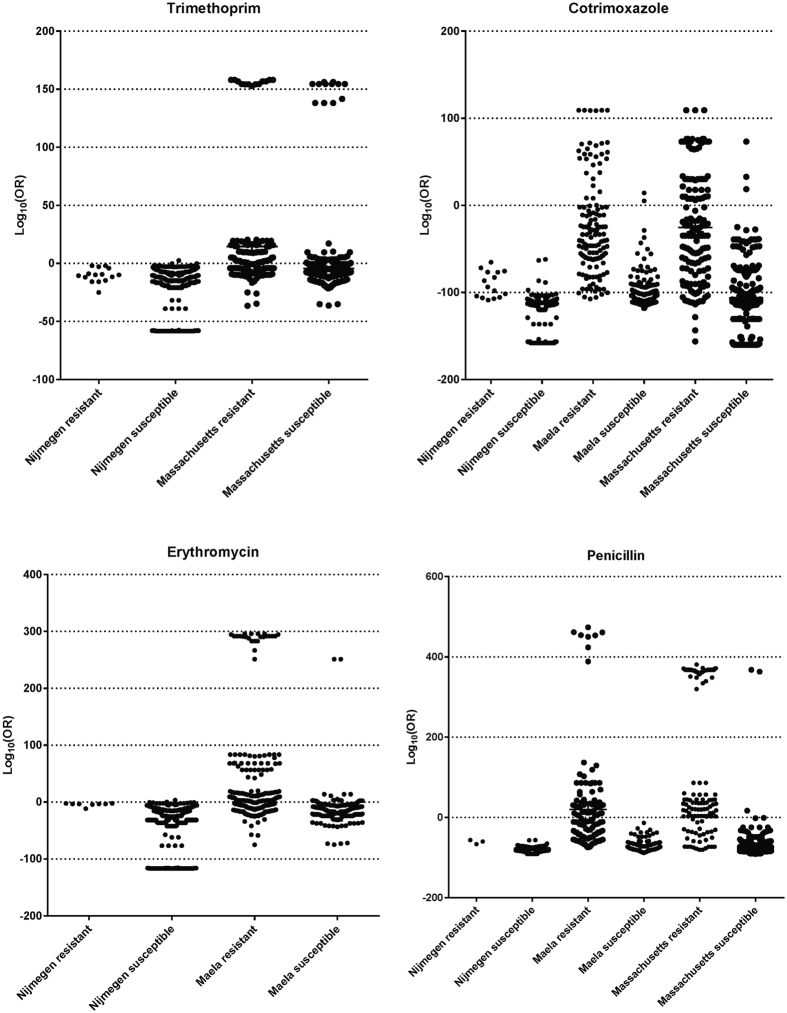
Penicillin, erythromycin, trimethoprim, and cotrimoxazole resistance profiles for isolates from individual geographical locations. Each point represents the cumulative odds ratio effect of SNPs that significantly associate with resistance on each isolate.

With a presupposition that accumulation of particular SNPs, or acquisition of certain genes leads to a creep towards antibiotic resistance and subsequently full-resistance, we sought to categorize the strains according to their phenotype of antibiotic resistance using the putative resistance-conferring SNPs and/or genes identified by a GWAS. We identified the partition between isolates by summing up the logarithmic derivatives of the odds ratios (*s*OR) for the putative antibiotic resistance-conferring SNPs (Fig. 3).

**Figure 4:**
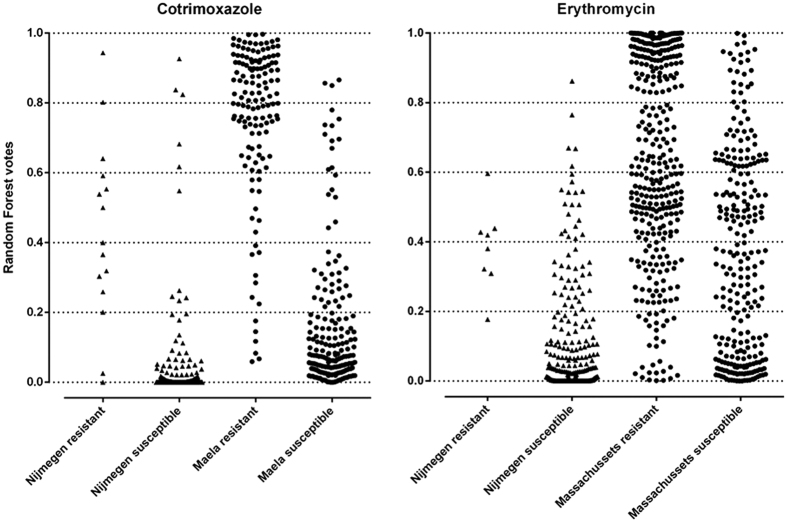
The difference in resistance profiles between invasive and carriage isolates. Each point represent the cumulative random forest vote for invasive (triangles) and carriage (circles) isolates.

**Figure 5:**
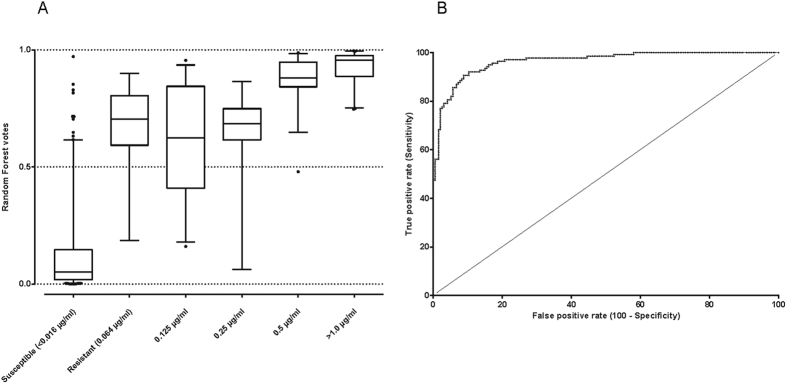
(**A**) A box whisker plot of the random forest votes for the levels of resistance to penicillin. Increase in resistance (MIC >= 0.06 µg/ml), as measured by relative increase in the MIC per isolate, is coupled with increased accumulation of the resistance-conferring SNPs that in turn uplift the votes. (**B**) A Receiver Operating Characteristic (ROC) curve showing the discrimination power of this random forest model in predicting penicillin resistance (Area Under Curve; AUC min = 0.9417 and max = 0.9882 at 99.9% CI).

In response to environmental stress over time, bacteria evolve genetic adaptations such as the acquisition of resistance genes or accumulation of critical mutation that confer antibiotic resistance. Supplementary Figure 1 shows the resistance profiles for four antibiotics over time in different cohorts. There seems to be an increase, however subtle, in the number of isolates that have accumulated more resistance-conferring SNPs in each cohort every year. The IPD isolates from Nijmegen have particularly accumulated combinations of SNPs that confer resistance but are more close to susceptibility as compared to carriage isolates. From this data, it is possible to model the resistance profile of new clinical isolates and to predict strains that are approaching extreme antibiotic resistance using sequencing-based approach. Using the RF analysis, we observed that carriage (non-invasive) strains exhibited more resistance to antibiotics (through accumulating SNPs acquiring genes that are most likely to) than invasive strains (Fig. 4). They mostly contained resistance-associated SNPs with high odds ratios of conferring resistance. We also observed that increase in minimum inhibitory concentrations (MIC) against penicillin is characterized by an increase in high *s*ORs SNPs are prioritized by the RF model (Fig. 5). Additionally, we observed that the antibiotics-susceptible isolates (MIC < 0.016 μg/ml) have also acquired some level of mutations that bring them closer to low-MIC penicillin resistance, which in clinical practice can be managed by increased penicillin dose.

## Discussion

This study includes a total of 1,680 *S. pneumoniae* isolates from diverse cohorts. Of these, a total of 1,012 carriage isolates (Massachusetts and Maela), and 350 IPD isolates (Nijmegen) were included in a GWAS to pre-screening for resistance-conferring variants. Another 318 SCD isolates were later included in the post-association evaluation of SNPs to determine the distance to resistance. Compared to the general population, SCD patients are usually at high risk of contracting potentially fatal IPD. Therefore, they receive long-term antibiotic prophylaxis and frequent empiric antibiotic treatment. In response to the antibiotic selective pressure, pneumococci isolated from SCD patients have been shown to exhibit high rates of antibiotic resistance^10^. As a result, isolates from these patients were expected to exhibit substantial resistance that could skew the statistical associations.

Antibiotic resistance profiles differed significantly between sampled populations, and between invasive and nasopharyngeal carriage isolates. Rates of antibiotic resistance were generally low in isolates from the Netherlands compared to those from Thailand and USA. The low levels of resistance in the Netherlands could be explained by two reasons. First, all Dutch isolates used in this study are from IPD cases whereas the Thai and US isolates are predominantly carriage isolates. There is ample opportunity for pneumococci in carriage to benefit from multiple co-colonizing strains of same or closely related species that provide new genetic material for homologous gene transformation. Rates of pneumococcal transformation are also much higher during colonization than during planktonic growth in sepsis^16^. Second, the Netherlands has implemented very stringent antibiotic control policies. The WHO provides defined daily dose (DDD) guidelines for antibiotics use in adults. Research has shown that aggregate hospital antibiotic use by DDD in the United States is discordant with the WHO stipulations^17^. Most studies established that majority of antibiotics were administered in primary care settings to treat infections for which antibiotic therapy is hardly indicated^18^,^19^. Similarly in Thailand, antibiotics are readily sold over-the-counter allowing for endemic misuse^20^. Such trends are likely to have accelerated the pneumococcal selection for resistance in the US and Thai populations, and could explain the observed higher levels of resistance as compared to, for example, the Dutch population where antibiotic use is vastly prudent.

Penicillin and other β-lactams have for long been the primary means of treating pneumococcal infections and are perhaps the most widely used group of antibiotics that work by inhibiting bacterial cell wall biosynthesis^21^. Resistance arises when the organism produces beta-lactamases, which are enzymes that cleave the beta-lactam ring, or modifies the drug targets; ‘penicillin binding proteins’; PBPs. Published research implicates changes in PBPs as the primary determinants of β-lactams resistance^22^,^23^,^24^. Variations in PBP2b and PBP2x modulate low-level (intermediate) β-lactams resistance with additional changes in PBP1a leading to extreme resistance. Growth inhibition assays show that β-lactams primarily kill the pneumococcus by inhibiting PBPs, particularly PBP2×^25^. PBP transpeptidase signatures are also significant indicators of resistance levels in various β-lactams^26^.

SNPs associated with beta-lactam resistance were previously reported using GWAS^6^. We identified 4,317 penicillin-resistance associated SNPs in the pneumococcus, which is more than Chewapreecha and colleagues observed (858 and 1,721 in Maela and Massachusetts cohorts respectively - 301 common SNPs). The difference could be explained by the fact that Chewapreecha *et al*. replicated their statistical associations in two independent cohorts and only collated candidate SNPs that were identified to be common between the two groups. In contrast, we selected all SNPs showing significant statistical association (*p*-value < 0.01) with the phenotype after correcting for multiple testing. Moreover, we potentially introduced more genotypic variance by analyzing a mixture of isolates, especially the disease isolates, from different geographical areas. The divergent genotypes in practice resulted into more sequence clusters (used for population substructure stratification) further partitioning the phylogenetic clusters/clades that may have been considered similar in the Chewapreecha study. Therefore, our approach allowed for identification of more SNPs. Applying a more stringent *p*-value threshold, however, reduced the number of SNPs allowing for further prioritization using RF. The onset of these SNPs is perhaps indicative of the development of antibiotic resistance. However, more studies will be required to validate these observations.

In the case of penicillin resistance, a single SNP or gene is not enough to confer full resistance. We used the RF analysis to evaluate the contribution of combinations of SNPs and/or genes to resistance to penicillin. The mixed RF model incorporating SNPs and genes showed that resistance to β-lactams is primarily driven by mutations in key genes and possibly by the presence of certain resistance genes. In this model, only the *gpsB* gene was among the top penicillin-resistance conferring features. GpsB is thought to be putatively essential^27^ and thus expected to be present in a single copy in all isolates. however, there appears to be two variants of the *gpsB* gene, og_1645; n = 1457 and og_2891; n = 215 in our isolates. GpsB is vital for peripheral and septal peptidoglycan synthesis in *S. pneumoniae*, particularly in the recruitment of PBP1 to the division complex and its removal from the cell pole soon after pole maturation is completed^27^. It shows overlapping although non-identical pattern of co-localization with FtsZ during cell division. Depletion of *gpsB* causes division defect characterized by significant cell elongation and enlargement, several unconstricted rings of division proteins Pbp2x, Pbp1a, FtsZ, and MreC, cessation of growth, and eventually cell lysis in *S. pneumoniae* D39^27^. These phenotypes are similar to those observed in Pbp2x depletion^28^ or inhibition of Pbp2x by the β-lactam antibiotic methicillin^27^. Therefore, the observed association is most likely a secondary effect of the functions of gpsB in peptidoglycan synthesis during cell division which may have fitness and pleiotropic consequences in maintaining cell integrity rather than a direct role in resistance.

Trimethoprim and cotrimoxazole (a combination of trimethoprim and sulfamethoxazole) reduce the ability of some bacteria to utilize folic acid for growing^12^ by blocking folate metabolism via *dhfR* or *dyr* (encoding dihydrofolate reductase), and *folP*/*sulA* (encoding dihydropteroate synthase) respectively. Although there is increasing prevalence of resistance of *S. pneumoniae* to these drugs, they are still deemed as excellent treatment particularly for pneumococcal infection as well as more generally for otitis, sinusitis, and acute exacerbation of chronic bronchitis. Mutational or recombinational changes on the target enzymes; *dhfR* and *folP* or their promoter regions have been reported to enhance resistance to trimethoprim and cotrimoxazole^12^,^13^,^29^. As expected, SNPs in various genes of the folate metabolism pathway correlated with resistance to trimethoprim and co-trimoxazole. Other significant associations for SNPs in genes implicated in resistance to other essential antibiotics, for example PBPs (*pbp1A, pbpX, penA*) implicated in penicillin resistance, indicating a strong co-selection for resistance to different classes of antibiotics by the pneumococcus. This could be due to the frequent simultaneous use of these antibiotics or an epistatic effect of linked compensatory pathways. More studies will be required to validate this observation.

Macrolides remain an important class of antibiotics for pneumococcal disease^30^. They inhibit protein synthesis by penetrating the bacterial cell membrane and binding to the ribosomal RNA molecules, particularly in the 50S subunit, of the bacterial ribosome blocking the exit of the growing peptide chain. Their prevalent use in other indications may be the primary driver of selection for macrolide resistance in pneumococci^31^,^32^,^33^. Pneumococcal resistance to macrolides is caused by drug efflux or alteration of the target site^34^,^35^. The phenotypic expression of target-site modification can be inducible or constitutive, and can be confirmed with the presence of *mefA*/*E* and *ermB* genes. *MefA* and *mefE* share >90% sequence homology and are carried in transposons which are comprised of additional open reading frames^36^. Perhaps due to the low prevalence of *ermB* (n = 124), the significance of statistical association waned after adjusting for multiple testing. Nonetheless, presence of other proteins like ImpB, YolD-like protein, SP_1114, and the macrolide efflux pump (encoded by *mefA*) was determining of resistance to erythromycin. The functional protein interaction network STRING shows that SP_1114 directly interacts with various efflux pumps associated with antibiotic resistance: the drug efflux ABC transporter ATP-binding protein/permease (SP_1342), the MATE efflux pump (SP2065), and the MATE family DinF transport system (SP1939)^37^, which may suggest a possible contribution to the efflux of drug. This may, however, need more studies to substantiate. YolD-like proteins, like their presumed equivalent (UmuD) in Gram-negative bacteria^38^, could be involved in modifying the DNA replication machinery to allow bypass synthesis across a damaged template. ImpB on the other hand copies undamaged DNA at stalled replication forks which arise *in vivo* from mismatched or misaligned primer ends. These misaligned primers could be extended by DNA polymerase IV subunit (polIV). The important role of these proteins in division and maintaining cellular integrity may perhaps explain their indirectly contribution to erythromycin resistance.

Fluoroquinolones (Ofloxacin and ciprofloxacin) are a family of related compounds that inhibit bacterial DNA synthesis by promoting cleavage of DNA in the DNA-enzyme complexes of DNA gyrase and type IV topoisomerase. Ciprofloxacin binds exclusively with topoisomerase IV whereas ofloxacin binds more avidly with topoisomerase IV and also binds the gyrase. Fluoroquinolone activity in Gram-positive bacteria, including *S. pneumoniae*, usually results from inhibition of DNA type IV topoisomerase whereas activity in Gram-negative bacteria corresponds with inhibition of DNA gyrase. Pneumococcal resistance to fluoroquinolone is thought to be a stepwise process; most intermediately resistant strains accumulate first-step mutations, which usually involve only a single mutation in the target genes^39^, although, these strains do tend to go on to develop subsequent second-step mutations which significantly diminishes the activity of most fluoroquinolones and renders the strains highly resistant^40^. This increasing mutational heterogeneity makes it harder to use GWAS in precisely identifying the role of each mutation in disseminating antibiotic resistance to fluoroquinolones. While there were no significant associations for mutations in the target proteins, mutations in other genes like *pmrA, ccmA*, and *rarA* were observed to be determining of resistance to fluoroquinolones. Mutations in correlated with resistance to ofloxacin. PmrA is homologous to other well-studied efflux pumps like NorA and Bmr, whose expression leads to reduced susceptibility against several diverse compounds^41^, and is associated with fluoroquinolone resistance in the pneumococcus^42^. The pneumococcus, like most pathogenic bacteria, mainly uses iron from hemoglobin and heme to support growth^43^. The direct role of CcmA in quinolone-resistance is unclear but in Group A streptococci (GAS), the heme exporter confers multi-drug resistance^44^. Apart from the ABC-transporter protein PmrA^42^, efflux mechanisms of fluoroquinolone resistance are poorly characterized in the pneumococcus. Nonetheless, efflux could reduce intracellular fluoroquinolone concentrations to sublethal levels facilitating resistance development^45^. More experiments are required to deduce the exact mechanisms involving these other efflux pumps.

Recombination is the primary source of genome plasticity in bacteria. Unlike in β-lactam resistance where recombination plays an important role^46^, fluoroquinolone resistance arises from very specific resistance-determining mutations within the target proteins^47^. Horizontal transfer of fluoroquinolone resistance loci between viridans group streptococci and the pneumococcus has been shown to occur *in vitro* but not *in vivo*^48^, with significantly higher rates during asymptomatic carriage than during invasive isolates^49^,^50^. Studies have reported a link between fluoroquinolone resistance and evolution of resistance to penicillin and macrolides^51^; both of which could be fostered by recombination. These studies suggest that the observed association between ciprofloxacin resistance and mutations in *rarA* could be an artifact of the linked resistance with other antibiotics. However, since we cannot rule out a novel mechanism involving these mutations, more studies are required to ascertain this observation.

Pneumococci exhibiting decreased susceptibility to regular penicillin doses are on the rise^14^. Many European guidelines recommend high-dose empirical treatments for systemic infections caused by such strains showing atypical susceptibility. Analyzing the strains for an effective dose, however, relies on laboratory assays. A recent publication reports the use of PBPs transpeptidase signatures (TPDs) from 2,528 clinical pneumococcal isolates to predict the MICs for various β-lactam antibiotics^26^. Li and colleagues constructed predictive models that link amino acid sequence variations in the TPDs of PBP1a, PBP2b, and PBP2x to β-lactam MIC levels among invasive pneumococcal isolates. They identified 68, 78, and 118 unique TPD amino acid sequences for PBP1a, PBP2b, and PBP2x, respectively. Using 307 unique combinations of these sequences which defined the PBP types, they observed that isolates whose PBP types exhibited more than 10% amino acid sequence divergence from a usual susceptible PBP type were associated with increased β-lactam MICs.

Using GWAS and RF, we have rapidly detected decreasing sensitivity of the pneumococcus to increasing doses of penicillin, and we have classified susceptible and resistant isolate using genome sequencing with the sensitivity and specificity comparable to the MICs determined using phenotypic susceptibility tests. The increase in MIC corresponded with accumulation of SNPs and acquisition of genes that we prioritized to be most determining of penicillin resistance. Moreover, we have determined how far the antibiotic susceptible strains are from acquiring the resistance-conferring features that will enable them attaining full-resistance, and what the creep pattern towards full-resistance is. The measure is dubbed the “distance to resistance”. Starting with a curated reference database of genes and SNPs/alleles that confer resistance in historical and contemporary isolates; it is possible to model a framework that predicts the antibiotic resistance profile of an isolate, and project the transition towards resistance over time. Although causality cannot always be drawn from statistical association, bacterial GWAS provide the means for identifying genomic variants underlying important microbial phenotypes like antibiotic resistance. We have demonstrated that prediction of advancing antimicrobial resistance could be achieved *in silico* using genomic sequencing data. Therefore, this study invokes a change of perspective for future research to focus on not only identify genetic variants underlying the resistance phenotype but also detecting how these variants herald the advancement towards full-resistance. Such sequencing-based frameworks are not only affordable and consistent but also allow for simultaneous discovery of other essential pneumococcal features such as serotype and sequence type. Altogether, this knowledge will greatly inform the choice of clinical intervention and improve public health surveillance thus precluding outbreaks caused by emerging multidrug resistant strains.

## Materials and Methods

### Strains and phenotypes in study

This study included 1,680 pneumococcal isolates and corresponding antibiotic resistance phenotypes; 349 from adults admitted with invasive pneumococcal disease between 2001 and 2011 in two hospitals in Nijmegen, The Netherlands^8^, a systematic selection (three from each “secondary BAPS” cluster) of published carriage isolates from Massachusetts, USA and Maela, Thailand^6^, and 318 isolates from children suffering from sickle-cell disease (SCD) in the USA^10^ which included isolates from the CDC ABC bacterial surveillance core and published collections^52^,^53^. Phenotypes of antimicrobial susceptibility were determined *in vitro* as previously described^6^,^8^,^9^. For the invasive Nijmegen isolates, the following breakpoints were used: penicillin susceptible (S) = <00.6; co-trimoxazole S = <1; erythromycin S = <0.25.

### Determining SNPs and orthologous sequences

Bases were called from mapped sequences using kSNP v2 software^54^ against a single reference genome: multidrug-resistant *S. pneumoniae* ATCC 700669; Spain 23F ST81^55^. A total of 124,310 SNP calls were generated (Numerical feature IDs used in this study correspond to the SNP base-pair position in this reference genome). Filtering for SNPs present in more than ~90% of the isolates, (1,500 strains) resulted in 76,429 SNP calls that we used for further analysis. To determine clusters of orthologous sequences, all coding sequences (CDS) from the 1,680 isolates were predicted using Prodigal^56^. All coding sequences were analyzed using USEARCH^57^ and aligned in the ‘large-scale BLAST score ratio’ (LS-BSR) pipeline^58^ allowing 10% amino acid difference within clusters. The resulting representative sequences per group (“centroids”) were clustered through a Markov Clustering Algorithm (TRIBE-MCL)^59^ with an inflation factor of 2.5, resulting into 4687 orthologous groups (OGs). For each OG, we generated a binary metric of the presence (1) or absence (0) of a representative coding sequence(s) (CDS) from each strain. Each strain’s contribution of CDS to an OG was subsequently denoted by a single numeric value (1 or 0) to designate the presence or absence of a distinct gene, gene variants, or a group of paralogs. These groups were collated into a binary matrix and formatted for PLINK association analyses^60^.

### Determining the population structure and statistical association

Resistance phenotypes were grouped according to antibiotic classes and analyzed separately for each population, and together in the final association analysis. The population clusters used to control for the effect of clonal inheritance of genetic variants and population stratification were determined using the Bayesian Analysis of Population Structure (BAPS) software^61^, and a phylogeny-based partitioning approach as proposed by Prosperi *et al*.^62^, which employs ‘*ape, geiger, igraph*, and *phytools’* packages in R software^63^. An alignment of concatenated SNPs from the core or non-repetitive DNA of each of the isolates were analyzed in BAPS as previously described^8^. For the Prosperi clustering, a maximum-likelihood phylogenetic tree was constructed using RAxML version 8.2.0^64^ and an alignment of all the concatenated SNPs from the core genome of all isolates as described before^8^. The general time-reversible model was used to calculate the maximum-likelihood ratios with a γ adjustment for site variation as the nucleotide substitution model. The support for nodes on the tree was tested using a hundred unsystematic bootstrap replicates. Resulting phylogenetic tree was visualized using iTOL version 2.1^65^.

We used the Cochran-Mantel-Haenszel (CMH) correlation statistic to test for associations between antibiotic resistance phenotype and SNPs conditional on the population structure. Stratification for population structure minimized falsely positive associations that could be obtained merely by chance. We tested associations for resistance to penicillin, trimethoprim, cotrimoxazole, erythromycin, ofloxacin and ciprofloxacin, and corrected for population stratification using the genetic subpopulations (represented by the sequence clusters; SCs) determined using BAPS and/or the method proposed by Prosperi *et al*.^62^. The statistical associations were performed using PLINK software v1.9^60^, and the results visualized as Manhattan and Q-Q plots in R using ‘*qqman*’ package.

### Candidate resistance loci and a measure of the distance to resistance

We selected SNPs showing statistically significant associations (*p-values* < 0.01 at a minor allele frequency >0.01; Bonferroni-adjusted for multiple testing) as candidates for subsequent analysis. The percentage distribution of these candidate SNPs within resistance isolates relative to the susceptible isolates in each population was computed to determine how they vary in each cohort. For each SNP significantly associated with antibiotic resistance, we determined the odds ratio (OR) and nature (positive or negative) of the correlation. The accumulation of these significant SNPs in each isolate was also defined across all test cohorts. Each SNP present in an isolate was represented by the logarithmic derivative of the odds ratio; log_10_ (OR): The negative logarithmic values were used for SNPs negatively correlated with resistance. Q-Q plots were used to determine a more stringent *p-*value cut-off. The aggregate effect of the SNPs conferring antibiotic resistance is the sum of all the log_10_ (OR) values for SNPs above the *p*-value threshold. These represented a measure of the level of resistance of an isolate. These aggregate values were plotted in GraphPad Prism v6.05 software.

### Random forest analysis for prioritizing variants and classifying isolates

To prioritize probable causal variants and investigate the effect of SNPs and/or gene combinations, a Random Forest (RF) classification using the Bioconductor *randomForest* package 4.6–10 was performed. The resulting candidates were used to discriminate resistant (R) and susceptible (S) isolates. This classification model, consisting of 5000 decision trees was trained on candidate genes and/or SNPs that were determined to be predictive of resistance through GWAS analysis. Statistical significance of the genes or SNPs that were able to discriminate between the classes were calculated by permuting the sample class labels. A normal distribution was determined for mean decrease of accuracy (MDA) values from the 300 feature-vector permutations using the *pnorm* package which is part of the R version 3.3.0 distribution^63^. Still using the *pnorm* package, a p-value was calculated comparing the average of mda values for RF 100 analyses (the out-of-box - OOB - error averaged over 100 RF runs) with the original sample classes (to account for slight differences between RF analyses) to the distribution of permuted MDA values.

## Additional Information

**How to cite this article**: Mobegi, F. M. *et al*. Deciphering the distance to antibiotic resistance for the pneumococcus using genome sequencing data. *Sci. Rep.* **7**, 42808; doi: 10.1038/srep42808 (2017).

**Publisher's note:** Springer Nature remains neutral with regard to jurisdictional claims in published maps and institutional affiliations.

## Supplementary Material

Supplementary Figure 1

Supplementary Table 1

Supplementary Table 2

Supplementary Table 3

Supplementary Table 4

Supplementary Table 5

Supplementary Table 6

## Figures and Tables

**Table 1:** Single nucleotide polymorphisms that are associated with penicillin resistance.

BP Position	RF importance	RF *p*-value	CMH *p*-value	Annotation	Gene
1613086	20.62957785	0	3.308E-99	penicillin-binding protein 2b	*penA*
1612897	19.83961807	0	6.649E-85	penicillin-binding protein 2b	*penA*
333792	11.86365577	8.69E-98	1.234E-99	Holliday junction-specific endonuclease	*recU*
333282	11.70867819	1.55E-18	5.304E-55	penicillin-binding protein 1 A	*pbp1A*
334107	11.29654702	7.83E-162	9.151E-72	Holliday junction-specific endonuclease	*recU*
334639	10.42365322	0	1.203E-68	hypothetical protein	—
294991	10.24670847	0	5.276E-60	phospho-N-acetylmuramoyl-pentapeptide-transferase	*mraY*
335104	8.53526957	3.3E-17	2.659E-68	DivIVA protein	—
333345	7.379638353	6.46E-16	8.607E-24	penicillin-binding protein 1 A	*pbp1A*
1613422	7.320298527	9.51E-56	4.344E-55	penicillin-binding protein 2b	*penA*
292563	7.167180495	8.14E-08	1.294E-55	penicillin binding protein 2x	*pbpX*
332247	6.771832321	4.27E-26	3.671E-55	penicillin-binding protein 1 A	*pbp1A*
335955	6.631966798	8.25E-35	9.742E-45	RNA methylase family protein	—
1613770	6.108715366	7.27E-87	2.023E-78	penicillin-binding protein 2b	*penA*
333386	5.891738108	4.68E-13	8.607E-24	penicillin-binding protein 1 A	*pbp1A*
1532326	5.159428663	2.95E-11	4.143E-72	dihydrofolate reductase	*dyr*
1531915	5.154251817	1.21E-11	1.717E-20	ATP-dependent protease ATP-binding subunit ClpX	*clpX*
295737	4.619274035	1.67E-75	7.271E-49	ATP-dependent protease ATP-binding subunit ClpL	*clpL*
296748	4.473732826	4.15E-08	2.701E-36	ATP-dependent protease ATP-binding subunit ClpL	*clpL*
2193975	4.432372852	3.41E-61	8.905E-27	elongation factor Ts	*tsf*

**Table 2:** Single nucleotide polymorphisms and genes that correlate with cotrimoxazole resistance.

Feature	RF importance	RF *p*-value	CMH *p*-value	Annotation	Gene
1531915	12.06447624	0	3.65E-32	ATP-dependent protease ATP-binding subunit ClpX	*clpX*
293661	10.58122979	2.05E-112	1.3E-75	penicillin binding protein 2x	*pbpX*
267233	10.09698603	0	5.656E-82	GTP cyclohydrolase I	*folE*
1532245	9.521815356	0	2.616E-81	dihydrofolate reductase	*dyr*
1613086	9.414234472	5.99E-237	8.051E-62	penicillin-binding protein 2b	*penA*
264912	8.905643954	0	1.032E-75	dihydropteroate synthase	*folP*
1531651	7.923116586	0	4.595E-60	ATP-dependent protease ATP-binding subunit ClpX	*clpX*
265536	7.247622768	5.56E-86	3.638E-67	dihydropteroate synthase	*folP*
291557	6.341493841	3.03E-43	3.097E-61	cell division protein	*ftsL*
292017	5.849575748	7.35E-31	4.103E-50	penicillin binding protein 2x	*pbpX*
1532054	5.822732316	0	1.203E-28	hypothetical protein	—
1612897	5.726042635	8.3E-102	1.213E-49	penicillin-binding protein 2b	*penA*
262352	5.671693703	0	1.208E-38	—	—
263885	5.610620269	9.89E-132	7.608E-41	permease	—
332799	5.455168203	5.73E-56	1.758E-48	penicillin-binding protein 1A	*pbp1A*
262539	5.429029061	0	2.304E-33	—	—
263190	5.333550128	9.15E-144	4.673E-26	permease	—
1613770	5.331345531	3.4E-65	2.497E-51	penicillin-binding protein 2b	*penA*
291982	5.096429153	4.18E-34	3.797E-47	penicillin binding protein 2x	*pbpX*
335104	4.434933551	0.000000165	1.249E-40	DivIVA protein	—

**Table 3:** Single nucleotide polymorphisms and genes that correlate with trimethoprim resistance.

Feature	RF *p*-value	CMH *p*-value	Annotation	Gene
267970	4.39E-262	2.51E-13	2-amino-4-hydroxy-6-hydroxymethyldihydropteridine pyrophosphokinase	—
291557	4.19E-57	6.03E-15	Cell division protein	*ftsL*
292017	4.99E-52	2.89E-16	Penicillin binding protein 2x	*pbpX*
291982	9.37E-47	1.34E-16	Penicillin binding protein 2x	*pbpX*
cog_1652	3.01E-198	3.54E-08	ImpB/MucB/SamB family protein	—
cog_1312	1.79E-58	0.00000173	Macrolide efflux pump	—
cog_1379	4.39E-85	0.000000119	YolD-like protein	—
cog_791	3.88E-48	0.00000049	Ribose import ATP-binding protein rbsA	—
cog_2061	1.41E-90	0.000000119	Hypothetical protein	—
291286	3.39E-15	7.68E-17	S-adenosyl-methyltransferase MraW	*mraW*
290545	3.38E-26	6.7E-12	—	—
1558235	4.29E-19	4.69E-11	Cytidylate kinase	*cmk*
cog_1645	0	0.001967	Cell division initiation protein	—
cog_2891	0	0.000622	Cell division initiation protein	—
cog_182	1.49E-25	0.001306	Binding-protein-dependent transport systems inner membrane component	—
2002234	2.83E-28	6.6E-15	PTS system ascorbate-specific transporter subunit IIC	—
1459029	5.58E-30	4.56E-12	Asparaginyl-tRNA synthetase	*asnC*
887794	1.4E-27	4.56E-12	DNA polymerase III subunit epsilon	—
1800425	0.0000387	1.73E-21	DegT/DnrJ/EryC1/StrS family amino sugar synthetase	—

**Table 4:** Single nucleotide polymorphisms and genes whose presence correlates with erythromycin resistance.

Feature	RF importance	RF *p*-value	CMH *p*-value	Annotation	Gene
og_1652	7.180656298	0	3.701E-14	ImpB/MucB/SamB family protein	—
og_1379	7.175992284	0	6.409E-15	YolD-like protein	—
og_2061	7.097377018	0	6.409E-15	hypothetical protein	—
og_1312	6.485953794	1.71E-217	2.683E-14	Macrolide efflux pump	*mefA*
og_791	6.017699918	1.03E-210	6.671E-14	Ribose import ATP-binding protein rbsA	—
1741683	4.904707263	8.7E-52	3.01E-23	Nudix-related transcriptional regulator NrtR	—
og_243	4.114250972	0.00000718	0.0002367	IS1380-Spn1 transposase	—
og_10	3.24238261	0.000118	0.01343	3-ketoacyl-ACP reductase	—
290545	3.240769624	1.2E-76	6.7E-12	Tn916 ORF16 ATP/GTP-binding protein	—
297653	3.016787219	4.01E-28	2.24E-16	methyltransferase small domain superfamily	—
og_53	2.971456253	5.89E-142	4.8E-09	UDP-glucose 6-dehydrogenase	—
og_129	2.962230108	0.000564	4.443E-07	chlorohydrolase	—
678325	2.695815692	1.6E-53	2.15E-15	transposase, ISSmi4	—
136082	2.412872116	2.23E-15	7.73E-14	cytidine deaminase	*cdd*
637379	2.41015196	6.07E-44	1.36E-10	nucleotidyl transferase WchZ	—
987093	2.253968194	7.1E-12	4.43E-23	Abi-alpha protein	—
2058103	1.964208468	8.09E-10	5.51E-18	transposase	—
og_4190	1.95393053	0.385	0.4235	IS630-Spn1, transposase Orf1	—
1593956	1.93060019	8.68E-09	1.62E-10	Ribose import ATP-binding protein rbsA	—
794795	1.754564135	0.000000235	1.62E-10	hypothetical protein	—
