# Supplementary figures and images for: Deciphering the distance to antibiotic resistance for the pneumococcus using genome sequencing data
Source: Sci Rep. 2017 Feb 16;7:42808. doi: 10.1038/srep42808 (PMC5311915; doi:10.1038/srep42808)

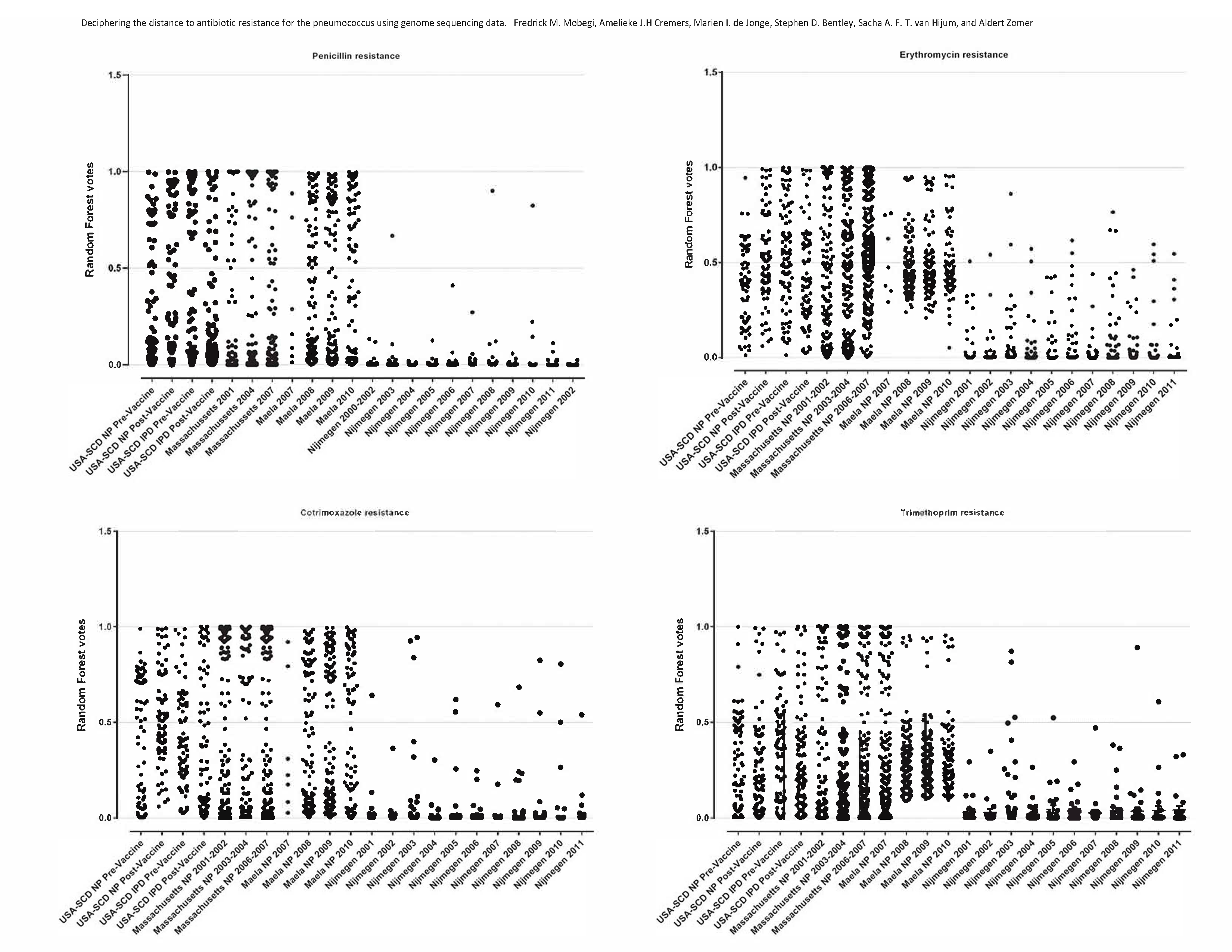

Supplement: Supplementary Figure 1 [file srep42808-s1.tiff]
